# TEN RILLINGTON PLACE AND THE CHANGING POLITICS OF ABORTION IN MODERN BRITAIN*

**DOI:** 10.1017/S0018246X14000399

**Published:** 2014-12

**Authors:** EMMA L. JONES, NEIL PEMBERTON

**Affiliations:** University of Manchester

## Abstract

This article addresses the social, cultural, and political history of backstreet abortion in post-war Britain, focusing on the murders of Beryl Evans and her daughter Geraldine, at Ten Rillington Place in 1949. It shows how the commonplace connection of John Christie to abortion and Beryl Evan's death was not a given in the wider public, legal, political, and forensic imagination of the time, reflecting the multi-layered and shifting meanings of abortion from the date of the original trials in the late 1940s and 1950s, through the subsequent judicial and literary reinvestigations of the case in the 1960s, to its cinematic interpretation in the 1970s. Exploring the language of abortion used in these different contexts, the article reveals changes in the gendering of abortionists, the increasing power and presence of abortion activists and other social reformers, the changing representation of working-class women and men, and the increasing critique of the practice of backstreet abortion. The case is also made for a kind of societal blind spot on abortion at the time of both the Evans and Christie trials; in particular, a reluctance to come to terms with the concept of the male abortionist, which distorted the criminal investigations and the trials themselves. Only when public acceptance for legalizing abortion grew in the more liberal climate of the 1960s and beyond did a revisionist understanding of the murder of Beryl Evans, in which abortion came to be positioned as a central element, gain a sustained hearing.

In 1971, *10 Rillington Place*, a film starring Richard Attenborough as the real-life serial killer, John Christie, opened in British cinemas.^1^ Shot on location, with meticulous detail and gritty social realism, the film focuses on the turbulent marriage of a young and gullible couple who live in the attic room of a rundown Notting Hill tenement in 1949. Undoubtedly, contemporary audiences would have been familiar with the history of this extraordinary house and case, because, for over two decades, Ten Rillington Place was a watchword for injustice, legal, and political corruption, deprivation, horror, and multiple murder in post-war Britain. The horrors of the Christie murders are recapitulated, not only to convey the sadism of a deranged serial killer, but also to depict the horrors of life in Britain under the shadow of repressive legislation and morality, a Britain struggling to break free from an intrinsically Victorian mould. The case is commonly recognized to have sounded the death knell of capital punishment, although it also gave evidence of the extreme dangers, which some women faced, in a country where the act of abortion was both illegal and stigmatized.

The film opens with the story of the relationship between Beryl and Timothy Evans. It follows the events that took place when Beryl, played by Judy Geeson, revealed that she was pregnant and told her husband that she wanted to ‘get rid of it’. Timothy, played by John Hurt, is portrayed as uncertain. At this point, Christie, their neighbour in the Notting Hill tenement, becomes an important figure in the narrative. Christie presents himself as a man who can help the couple: he boasts to them of his suitable medical credentials and so they decide to go ahead with the operation.

The subsequent scenes are harrowing. In his role as a pseudo-medical practitioner, Christie prepares Beryl for the abortion in her top floor flat of the tenement. He tries to make Beryl comfortable by giving her a cup of tea. Although obviously nervous, she begins to relax. She then removes her knickers and is directed by Christie to a mattress on the floor where she lies down. Christie moves over to her and places a mask over her face, through which he pumps carbon monoxide to induce unconsciousness, telling her this will help with her discomfort. Due to the highly unpleasant nature of this experience, Beryl becomes distressed and starts to scream. To hold her down, Christie climbs on top of her. He begins to strangle her, and rapes her as she dies.

The film depicts Beryl Evans as being one of Christie's many victims, including the Evans's baby daughter, Geraldine. Yet in reality, Timothy Evans, not Christie, was convicted and hanged for his daughter's death in 1950, and was also widely believed to have been guilty of murdering his wife. Three years later, following the revelation that John Christie had killed at least six women, including his own wife Ethel, doubt was raised over the safety of Timothy Evans's conviction. While most popular and conventional accounts of the murders tend to focus on legal aspects of the case, this article directs attention to a side of the case that has been relatively overlooked: how the moral, social, and legal issues surrounding abortion and the illegal abortion trade were powerfully and influentially implicated in the circumstances surrounding Beryl's death, conditioning both expert and lay understandings of her demise.^2^

In modern-day popular understandings of the horrors of Ten Rillington Place, John Christie became the archetypal figure of the nightmarish backstreet abortionist, who, armed with rubber tubing and gas (his ‘anaesthetizing’ equipment), ‘did away’ with his unfortunate victims, who had come to him to get an abortion. In an era of liberalization, the imagery of the villainous male abortionist with ulterior sexual motives plays a significant role in the modern demonization of Christie as a predatory rapist; reminding us of an age when legislation meant that women were willing to risk serious injury or even death at the hands of such a monster. However, it is critical to note that the connection of Christie with abortion, made so apparent in the film, was not a given in the wider public imagination at the time of the murders. Neither in 1949 nor 1953 was abortion taken seriously by lawyers, reporters, or members of the public as an adequate explanation for the circumstances surrounding Beryl Evans's death.

This absence requires an appreciation of the historical and cultural factors shaping and shifting lay and expert understandings of abortion, and its place in public discourse. As we will demonstrate, the positioning of abortion as one of the central narrative frameworks in Beryl Evans's death came about through the new public discourse of social morality, which developed during the late 1950s and 1960s. This led to a broad interrogation of the interrelationships between morality and law, creating a context conducive to a series of liberalizing reforms concerning capital punishment, marital relations, and sexual behaviour, all of which were indicative of a relaxing of public and private mores. As part of this, abortion was made legal in 1967.

As historians of abortion have convincingly shown, the practices and politics of abortion were always about more than the act itself. Wider public understanding of abortion reflected cultural anxieties concerning social class, sexuality, race, and medical knowledge, and even permitted a range of voices to comment on the dangers of modern urban life and gender and family relations.^3^ Our preoccupation with the Christie case develops these analytical perspectives, but it also focuses historiographical attention on the gendering of the figure of the illegal backstreet abortionist, who in the collective cultural memory is most often thought of as a lay female occupation, being coded as naturally sympathetic and mothering, reflected most recently in the fictional figure of Vera Drake.^4^ By comparison, the figure of the male backstreet abortionist, which came to occupy a central place in the horrors of Ten Rillington Place, is a marginal one in both the collective memory and historiography.

In parliamentary discussion, reformist discourse, and in courtrooms, illegal abortion was overwhelmingly constructed as a ‘female crime’, with male abortionists rarely of central concern. However, when the male abortionist is discussed, he tends to be regarded as a more ambiguous, highly charged figure, whose activities were potentially more morally and sexually suspect. As Judith Allen provocatively argues, in representations of the abortion experience, male abortionists are presented as unsavoury and untrustworthy figures. The anxiety was that, in Allen's words, ‘men abortionists read the abortion situation as sexualized or erotically exploitable’.^5^ Drawing upon one detailed case-study, we expand this work by examining the figure of the male abortionist and the links between dangerous sexuality and backstreet abortionists, and the illicit practices surrounding the contexts, meanings, and encounters at Ten Rillington Place. We also show how these new imaginings of the male abortionist enabled contemporaries to rework narratives of the events that took place, carrying profound legal and political implications. In order to reconstruct the complexities of this subtext to the interior and exterior life at Rillington Place, we turn to post-mortem reports, newspapers, parliamentary sources, police records, abortion reform pamphlets, and trial transcripts, in order to recover the ambiguous meanings inscribed into the figure of the male abortionist.

The dynamic interplay between Ten Rillington Place and reproduction control also reminds us how public morality shaped what could be said about abortion, shrouding backstreet abortion in silence and secrecy, often with drastic consequences for the lives of those women who found themselves in court. Indeed, as a number of historians have convincingly revealed, abortion features prominently in the retelling of some of Britain's most infamous twentieth-century murder trials and executions, including those of Edith Thompson (executed, alongside her lover Frederick Bywaters, for the murder of her husband in 1923) and Ruth Ellis (hanged in 1955 for the murder of her lover David Blakely).^6^ As Annette Ballinger has observed, in both trials, the fact of abortion remained well hidden. Neither woman was able to discuss her experiences openly in court, because they lacked the appropriate language to do so effectively. Moreover, they lacked an ability to communicate within the boundaries of the dominant modes of expression and gender ideologies of the courtroom, which dictated that women conform to ‘traditional expectations in the areas of sexuality, respectability, domesticity and motherhood’.^7^ However, whereas criminal abortion was considered ‘unspeakable’ in the courtroom, in fictionalized accounts it assumes a critical place: provoking sympathy for the accused, providing evidence of their wrongful conviction and punishment and eliciting commentary on sexuality, class, and gender in modern society. As a highly taboo issue, discursively structured and constrained by hegemonic gender and sexual ideology and moral codes, it only served to reinforce the borders between guilt and innocence, male and female, working class and middle class.

This article examines the multi-layered discourse of abortion within one case in particular, and discusses how it has subsequently shifted, reflecting changing legal and public understanding of experiences, practices, and cultural meanings of abortion. To show this, the article is therefore organized around four separate historical events: the 1950 Timothy Evans trial; the trial of John Christie in 1953; the 1965 Brabin Inquiry, which investigated whether John Christie, not Timothy Evans, was responsible for Beryl Evans's death and whether the wrong man had been executed; and the 1971 film discussed above. Weaving in and out of these four events are two interrelated abortion stories, each of which is also destabilized, questioned, and revised as events unfold, due to shifting legal, popular, medical, and social understandings of backstreet and medical abortions. The first concerns Beryl Evans, who was purportedly seeking to end an unwanted pregnancy; the second concerns her neighbour John Christie, who allegedly posed as an illegal abortionist.

While the two stories were present from the outset, they were held in tension, their validity being assessed against other competing versions of the events at Ten Rillington Place. As we will show, in each of these historical moments, the circumstances of the death of Beryl Evans and the identity of Christie and whether or not he was an abortionist, were both reinvestigated and repositioned within a broader dialogue on abortion. Later, the lives of Beryl and Timothy Evans, a couple expecting a second child, were revised repeatedly. Each reiteration, as we will show, produced new moral truths about not only the reputation of these two individuals, but also the nature of working-class marriage in general. Within these shifting evaluative judgements, sustained in courtrooms and police investigations, the stories of Ten Rillington Place often served as moral dramas and exposés, in which the reputation of abortion seekers, men as well as women, were ambiguously positioned, scrutinized, and judged.^8^ Simultaneously, the configurations of Christie as a pseudo-abortionist brought sexual and gender distrust to the fore, consequently transforming the male backstreet abortionist into a potential sexual predator. Read against the multiple horrors of Ten Rillington Place, the transaction between the pregnant woman and the male backstreet abortionist allowed for the expression of the full range of ambiguous representations of male and female sexuality and working-class life, and of the taboos and silences surrounding abortion. However, it also had a role to play in shaping initial assessments of Timothy Evans's guilt and of how Beryl Evans had died.

## I

In December 1949, the bodies of Beryl Evans and her six-month-old daughter, Geraldine, were found bundled together, concealed in the washhouse at a dingy tenement at Rillington Place, in the Notting Hill district of London.^9^ Both had been strangled. In the weeks that followed, the police charged Timothy Evans with the murder of his wife and child. Put on trial for the murder of his daughter on 11 January 1950, he was executed three months later, on 9 March.

During the police investigation, Timothy Evans made three confessions.^10^ The first of these narrated Beryl's death as a self-induced abortion that had gone wrong. In the second, Beryl was presented as the victim of a botched abortion performed by a third party, identified by Timothy as the couple's neighbour, John Christie. Finally, in Timothy's third statement he confessed to strangling his wife in a fit of temper, positioning Beryl as a victim of domestic violence. These three statements each suggested a different cause for Beryl's death, setting up the two discursive frameworks that would come to shape early understandings of the case, and compete for dominance and authenticity: abortion and domestic violence.

For a brief moment, and as a result of Timothy's first two statements, the investigation was driven by abortion. As was usual in suspected criminal abortion cases, the police conducted a careful search for instruments that could have been used to procure abortion, and other evidential signs that such a crime had taken place. Instruments would have included syringes, douches, and bottles containing medicinal ﬂuids, powders, pills, or ointments. Other evidential signs would have included stains on furniture, denoting that it may have been used as a makeshift medical couch. Firegrates and ashbins would also have been subject to searches for traces of the crime which may have been burnt by the offender.^11^

In the course of their investigations, police officers discovered a Higginson syringe belonging to Ethel Christie. An everyday household medical implement, easily purchasable from a pharmacy, the syringe was used by women to self-douche and by both sexes to cleanse and regulate the digestive system through enemas. However, it also evoked the social and cultural illicitness of abortion, as, when used with water containing soap or another caustic substance, it was transformed into a potentially dangerous abortion device. However, as it was old and broken, detectives quickly ruled out the syringe as potential evidence.

John Christie and his wife were also questioned regarding the allegation of abortion. As with other friends and family interviewed, they had been aware of Beryl's desire to terminate her pregnancy, but denied any involvement in assisting her. Indeed, the Christies told of how they had advised Beryl to stop taking pills and various other things with which to procure an abortion, on account of how ill she had begun to look.^12^ When the bodies of Beryl and Geraldine were discovered, and Timothy Evans confessed to both murders, abortion was quickly ruled out as the dominant explanation for what had occurred at Ten Rillington Place. Instead, Beryl's murder was treated as a case of domestic violence, an account that was corroborated by medical evidence and witness testimonies.

Although a post-mortem, conducted by Donald Teare, pathologist and lecturer in forensic medicine at St Bartholomew's Hospital in London, revealed a small bruise on Beryl's vaginal wall, he deemed this had most likely resulted from a failed attempt to self-induce a miscarriage.^13^ While it was known to be common practice for women to take various pills and abortifacient remedies in an attempt to restore menstruation, it was considered extremely rare for women to induce an abortion successfully via internal methods, such as with a syringe, without assistance, and without endangering their lives.^14^ Aside from the bruise, Beryl's womb yielded none of the expected signs of criminal interference by an amateur backstreet abortionist, which as an experienced pathologist Teare would have recognized.^15^ There were, for instance, no internal tears or infection, no traces of an air embolism, and no signs of blood poisoning or sepsis. Most tellingly, Beryl's uterus had not been punctured and the sixteen-week old foetus was still intact. Closing off the possibility of criminal abortion prevented complicating the legal case, and avoided many difficult questions about a practice that was deemed highly unsuitable for public discussion.

The highly visible injuries to Beryl's bodily exterior – bruising, signs of asphyxiation, and ligature marks – seemed to offer a more convincing interpretation of her death as one of violence at the hands of her husband. Witness testimonies elucidated a picture of life at Ten Rillington Place in which the Evans's marriage was a turbulent one, characterized by ‘frequent quarrels’ over Timothy's ‘lying, his associating with other women, and financial matters’.^16^ They also indicated a history of violence between the couple: as star witness, John Christie, testified: ‘Mrs Evans has told my wife and I on more than one occasion that he [Timothy] has assaulted her and grabbed hold of her throat. She said he had a violent temper and one time would do her in.’^17^ The problem of an unwanted child was hence treated as one of a number of ignitable issues in the relationship of the young couple; a minor one at that. In pronouncing a guilty verdict upon Timothy Evans, the court passed what was believed to be its final judgement, thereby confirming the official narrative of Ten Rillington Place as the site of family breakdown and domestic homicide.

## II

In March 1953, rooms on the ground floor at Ten Rillington Place were empty, as John Christie and his wife had apparently vacated them. Another tenant, after gaining permission from the landlord to refurbish the backroom, discovered a body in a concealed kitchen cupboard. This would be the first of six bodies found in and around the ground floor flat and its garden. Immediately, the tenant raised the alarm. On arrival, Scotland Yard decided to excavate the whole of Ten Rillington Place. In the police investigation that followed, there were attempts to resolidify connections between abortion, John Christie, and Beryl Evans, yet these remained tentative and fragile.

In the days that followed, detectives and other investigators transformed the house from one previously associated with a lethal case of domestic violence into a site of multiple violent murders, now publicly identified with the serial murderer, Christie, who had lived and killed there. Forensic investigations revealed that after rendering his female victims unconscious by administering poisonous carbon monoxide, Christie strangled the women, as well as subjecting them to a violent sexual assault.^18^ The trial that followed would project Beryl Evans back into the public and forensic imagination; with her body this time reconceptualized as a valuable evidentiary resource in the assemblage of, and search for, trace evidence in the context of recent findings.

As public speculation intensified, the press ran a constant stream of articles insisting that Beryl had also been a victim of Christie. In custody, Christie confessed to the murders of seven women in all, including Beryl Evans. In a statement made to police on 5 June 1953, he explained that in the process of assisting Beryl in committing suicide, he had twice attempted to sexually assault her, and eventually strangled her to death. His recollection of events was riddled with ambiguity:

She said she would do anything if I would help her. I think she was referring to letting me be intimate with her … I turned the gas tap on and as near as I can make out, I held it close to her face. When she became unconscious I turned the tap off. I was going to try again to have intercourse with her but it was impossible, I couldn't bend over. I think that's when I strangled her.^19^

Yet, at no point did Christie confess to having been, or to having posed as an abortionist.

Christie's legal team took the surprising decision of testing the veracity of Christie's confession, by re-examining Beryl's body. On 18 March 1953, at the Kensington Cemetery, the bodies of Beryl and her daughter were exhumed. Medical and scientific investigators had the task of reassessing their tragic deaths in relation to Christie's predatory attacks on his other victims, who, with the exception of his wife, were allegedly prostitutes. Francis Camps, the pathologist who led the forensic investigation into the Christie murders, examined Beryl's body and concluded that there had been no evidence of tampering with the foetus, which remained intact.^20^ Camps also confirmed the results of Teare's post-mortem examination, which had found no evidence of any abortion activity.

While the investigations carried out by forensic pathology continued to disassociate Beryl Evans's body from Christie, other aspects of the case now allowed new, implicit connections to be made between abortion and Ten Rillington Place, especially following the discovery of the bodies of several young women. As Frank Mort argues: ‘The[se] corpses evoked powerful negative images of the female body as putrid, confirming the strongest associations between sex and death.’ These women's bodies, as Mort suggests, were treated as representative of the world in which they had lived and worked, one perceived to be suffused with sex.^21^ The third body found in the boarded-up scullery, that of Rita Nelson, a recent migrant from Ireland, was discovered to have been six months pregnant, thereby compounding the association between pregnancy and female sexual activity in and around Ten Rillington Place.^22^ Reported in the press, this finding aligned Rita Nelson with Beryl Evans – who had also been pregnant – and tentatively and implicitly evoked the scene as one of backstreet abortion, in addition to being a site of sexual attack and multiple murder. Though the work of an illegal abortionist was not explicitly detailed in relation to the murders in the press, due to public constraints on this highly sensitive topic, readers would have been familiar with the trappings of a tragic case of criminal abortion: the domestic scene, a deceased pregnant woman, and the dangerous amateur abortionist. Yet there was no public discussion of whether Christie had posed as an illegal abortionist to trap his victims.

The ensuing police investigation re-evaluated Christie as a potential illegal abortionist, however, resuming lines of inquiry into his activities in the wider neighbourhood. Rumours that an abortionist was operating at the tenement had reportedly led to the property being placed under police observation for some time prior to Christie's arrest, but investigations had apparently drawn a blank.^23^ For officials, the Christie case brought into sharp relief the abiding way in which the police and other officials had repeatedly conflated prostitution with petty crime and a range of other sexually illicit practices, including pornography, street-walkers, brothels, and abortion.^24^

Embedded within the police investigations into Christie, his neighbourhood, and the sexual deviance of Notting Hill was the world of backstreet abortion, and the social networks that made the practice possible. This provoked the question of how Christie enticed and entrapped his victims. The police collected statements that shed light on Christie's methods; witnesses told of how he had passed himself off as someone who could help ‘women in trouble’.^25^ However, at his trial, Christie was also charged with killing his wife, significantly curtailing courtroom discussions of not only his murdering methodology, but also the allegation that he posed as an illegal abortionist. The higher prosecution rate of women for practising abortion at this time compared to men may also have helped to sustain a societal blind spot on abortion during Christie's trial, and a reluctance in particular to come to terms with the concept of the male backstreet abortionist.

Tellingly, pregnancy and abortion were almost a taboo in the defence's line of questioning. The issue of abortion, referred to euphemistically, consumed a mere fifteen lines in the transcript of Christie's trial. Christie denied any involvement in procuring abortion; and even depicted himself and his wife as caring neighbours who persuaded Beryl to stop taking ‘any more stuff’ to terminate her pregnancy.^26^ In his summing up, the judge, Justice Finnemore, endorsed Christie's version of events surrounding Beryl Evans's death. Taking the jury back to 1949, Finnemore stated:

Mrs Evans was pregnant, and she said she did not want to have a baby because of the trouble between them [between Beryl and Timothy Evans]. Christie and his wife tried to stop her taking stuff to get rid of it. He said he never tried to procure an abortion. Do you not remember the rather suddenly emphatic voice in which he said that? Then the day before 8 November, he went upstairs and found that she had tried to gas herself.^27^

Finnemore effectively buried Evans's story that Christie offered Beryl an abortion, and sanctioned that Christie was thereby not guilty of Beryl's murder.

Found guilty of his wife's murder, Christie was sentenced to death on 25 June 1953, with his execution planned for 15 July. Before his execution, however, a parliamentary inquiry led by senior barrister Mr John Scott Henderson, was set up to investigate the safety of Timothy Evans's conviction and to determine if Christie had lied about his role and knowledge of the circumstances surrounding the deaths of Beryl and Geraldine in 1949. Published the day before Christie's execution, the Scott Henderson report confirmed Evans's guilt in the murder of his child (and by association his wife), stating there were no grounds to suspect that there had been any miscarriage of justice. In the report, Scott Henderson refutes Christie's status as an abortionist:

There are undoubtedly talks about abortion. Many persons knew that Mrs. Evans was trying to get rid of her pregnancy. There were vague references to someone helping her, but I can find no evidence that Christie's name was ever mentioned in this connection. He may have talked to various people about abortions. I am satisfied that the police made the most careful investigation in December 1949, as to his activities in this connection. They could find no evidence that he was an abortionist and were satisfied that he was not. The most material fact in this connection is that no interference with Mrs. Evans' pregnancy was attempted.^28^

Neither the Scott-Henderson inquiry nor Christie's execution brought closure to the public controversy surrounding Ten Rillington Place. Over the course of the following decade, Christie's status as an abortionist was to assume new importance, in the context of public anxiety about the safety of Evans's conviction and changing attitudes towards abortion and the cultural related meanings to it.

## III

In the wake of Labour's election victory in October 1964, there was a renewed call for the incoming home secretary, Frank Soskice, to sanction a new inquiry. In June 1965, a committee was established to lead a public review with the High Court judge, Sir Daniel Brabin, appointed as chair. Held over thirty days, with the cross-examination of original witnesses, the Brabin inquiry considered all the evidential dimensions to the case, but a central role was accorded to whether or not Beryl had willingly invited Christie to the top floor flat to perform an abortion on her.

Unlike previous trials and inquiries into the murderous history of Ten Rillington Place, the Brabin Inquiry would focus on the issue of abortion from day one. Indeed, its members returned to 1949, and to Timothy Evans's claims that his wife had died as the result of an abortion. In so doing, this new investigation positioned the house and its residents within a dark narrative about the sinister world of the backstreet abortion, a world increasingly regarded as an urgent social problem and political issue.

In the years leading up to the Brabin Inquiry it had been the journalist and broadcaster Ludovic Kennedy who had made a series of concrete connections between abortion and Ten Rillington Place. Kennedy made his name campaigning against a number of injustices and against capital punishment, establishing a literary genre through which these cases became known. Kennedy's book, *10 Rillington Place*, published in January 1961, was the result of a year's painstaking review of the Evans and Christie cases. He presented a dense, detailed reconsideration of the facts, which took in a wide range of material: for example, Christie's childhood and early adult traumas and illnesses, Evans's mental state, and the circumstances surrounding his ‘confession’. In doing this, Kennedy sought to present Rillington Place as a site of botched police investigations, establishment arrogance, and as the home to a necrophiliac psychopath. Most alarmingly, the execution of Timothy Evans represented an unequivocal case of judicial murder. By introducing abortion into the story, Kennedy revised the facts and circumstances surrounding the deaths of Christie's victims, presenting some of them as typical working-class abortion seekers, while at the same time articulating the image of Christie as a fraudulent, dangerous abortionist. For Kennedy, the recasting of Christie as a dubious backstreet abortionist and Beryl Evans as an abortion seeker remained central to the argument that Timothy Evans had been wrongly executed.

Kennedy's weaving of abortion into the narrative of the Ten Rillington Place murders coincided with how parliamentary and public opinion was moving in favour of abortion law reform, a result of the broad relaxation of sexual and social mores, and the growing realization of the heavy toll which illegal abortion exacted on maternal health and mortality.^29^ Since 1952, the year before the discovery of the bodies at Rillington Place, no less than six abortion bills had been proposed in parliament and the House of Lords; and in March 1965, a national opinion poll showed a two-thirds majority in favour of legalizing abortion in some cases. That year also witnessed the screening of Ken Loach's BBC television adaptation of Nell Dunn's *Up the junction* (1963), as part of the *Wednesday Play*, in which one of the working-class characters undergoes an illegal abortion. Combining drama and documentary, the show revealed how such backstreet operations were both dangerous and commonplace.^30^ Also in 1965, an episode of the popular medical drama, *Dr Finlay's Casebook*, featured an episode dealing with illegal abortion.^31^ Such progressive programming brought the social, legal, and moral issues of abortion to a wider public audience, and is credited with increasing support for the reform of the punitive abortion law.^32^

In his portrayal of how the murky and fraudulent world of backstreet abortion trade interwove with the Ten Rillington Place murders, Kennedy similarly blended various journalistic styles with melodrama, to circulate a powerful populist narrative of murder, darkness, urban decay, and injustice. Kennedy, for example, cast Rita Nelson as the epitome of an ‘abortion tourist’ explaining how she moved to London from Ireland three months pregnant, probably seeking to get rid of her baby.^33^

It is therefore not just possible but extremely likely, that Christie, meeting her in one of those squalid cafes where he used to go and look for sad women, learnt about her troubles and said to her, as he said to Mrs Evans, that he knew all about abortions and could help her out of her difficulties.^34^

Kennedy also made dramatic use of another woman, Margaret Forrest, who in 1953 was named in the press as the ‘woman who got away’ from Christie. According to Kennedy, the Forrest story provided firm witness testimony that Christie posed as a doctor who had been ‘struck off the medical register for doing a friend a favour’, and who now ‘lurked’ around the roadside cafes of west London, offering to help women with their ‘ailments’. To Mrs Forrest, Christie had intimated that he could perform abortions, which was ‘a lie’, Kennedy argued, ‘designed both to puff up his own ego and impress by laying claim to a knowledge that he didn't possess’.^35^

Part of the appeal of Kennedy's account – most notably, his commentary on the network of an unsafe abortion trade in a socially dilapidated West London – was how it came to echo the concerns of and rhetorical strategies deployed by abortion law reformers. Beginning in the 1930s, the political lobby for abortion law reform had focused on demanding access to safe, surgical abortions, and their arguments were laced with the language of class.^36^ Many proponents were motivated by a desire to remove the unjust division between the ‘unenlightened poor’ forced to undergo dangerous operations at the hands of unprofessional abortionists, and those who had the ‘knowledge, influence and financial resources' to obtain a secret but safe surgery performed by a doctor.^37^ The Christie case, in contrast, opened up a cultural space that fostered a distorted dialogue on the dangers of illegal abortion, through which the image of the ‘professional’ male abortionist was reversed to assume a menacing, sinister profile. It inadvertently revealed another, less vocalized side to the threat posed by the current ambiguous state of the law; the potential for deception, exploitation, and sexual danger embodied in the figure of the male backstreet abortionist. That Christie had posed as a backstreet abortionist with medical qualifications was, for Kennedy's revisionist argument, crucial. In his analysis, Christie had persuaded Beryl that he was capable of carrying out an abortion. Indeed, Kennedy now offered a new, compelling narrative of the events on the top floor of the flat, in which Beryl was under the impression that she was going to have an abortion performed by her middle-aged neighbour from the ground floor flat.

It was probably towards the end of the morning – Christie himself said about lunch time – that he mounted the stairs to the little kitchen where Beryl was waiting. She was dressed in a spotted cotton blouse, a light blue woollen jacket and a black skirt. She had probably removed her knickers in preparation before his arrival. According to Christie, ‘she brought the quilt from the front room and put it down in front of the fireplace. I am not sure whether there was a fire in the grate. She lay on the quilt. She was fully dressed.’ It seems almost certain that Christie, remembering the success he had had in gassing Eady [Murial Eady, another of Christie's known victims], had brought with him his piece of rubber tubing, hoping to persuade Beryl that if she took a sniff or two of gas, the abortion would be a great deal less painful … Whatever happened next must have happened very quickly. The most likely sequence of events is that Christie inserted either his finger or, for the sake of greater conviction, some blunt instrument like a spoon into Beryl's vagina, that almost simultaneously Beryl got into a wild panic, thrust against the rubber tubing and began struggling, that Christie, now in a state of frenzied sexual excitement and seeing his victim about to slip away, began hitting her savagely in the face and when she was semi-conscious, whipped out what he called ‘my strangling rope’ and strangled her.^38^

In his text, Kennedy went on to present Christie as a man locally known as an abortionist. On frequent occasions in cafés and bars, Christie posed as a doctor, earning him the nickname, ‘the Doc’; he spoke knowingly of medical matters and even offered to assist ‘women in trouble’. By calling upon gothic and melodramatic conventions, even invoking the Jekyll–Hyde dualism, Kennedy depicted Christie as a dangerous man with two personalities. In one guise, he was a polite, respectable and reserved man who had been a policeman during the war. In the other, Christie haunted the streets, seedy bars, and cafes in the nocturnal hours, disguising himself as a backstreet abortionist so he could entrap his victims.

Hyde [Christie] invariably answered the door at 10 Rillington Place, whether the caller was for him or not; when he let someone else answer the door, he watched the caller arriving through a little peep-hole he had bored in the wall above the kitchen door. Hyde was at home quite a lot; for he had wrangled certificate out of certificate out of Dr Odess to say he was unfit to work. Among his complaints were headaches, flatulence, diarrhoea and piles; the illnesses were as attractive as the man, often though Hyde was out especially in the evenings he wore plimsoll shoes so he could slink about unseen, his own shadow in a world of shadows, down among the con men and tarts and petty thieves. They too were impressed by his intelligence and his education, his gift of the gab. To them also he spoke of his medical knowledge but in rather different terms. He knew, he said, how to help young girls in trouble, indeed he had helped them in the past. The boast was never put to the test, but it helped to increase his standing. Ah, they said, he's a dark one, knows how to do abortions and all that.^39^

In Kennedy's depiction of Christie's murderous and monstrous self, he deploys the image of the backstreet abortion to illuminate a hidden world of danger, death, and decay in and around Notting Hill.

Though without the sensationalistic registers, the Brabin Inquiry also returned to the original witness statements, which now provided new, corroborative information alluding to Christie having posed as a ‘professional’ abortionist with medical credentials. According to these accounts, which complemented and extended Kennedy's earlier portrait of a serial killing abortionist operating in Notting Hill, Christie cut a convincing figure as a doctor, who, having fallen foul of the law, had resorted to helping women in difficulty. Witnesses spoke of a man fitting Christie's description – tall, well dressed, with horn-rimmed spectacles – who frequented the local bars and cafes of the neighbourhood, and approached women sitting inside, asking them about their ‘troubles’ and offering his services. More than one witness testified to this man telling them that he had been struck off the medical register for ‘helping girls out of trouble’. The same man boasted about his medical credentials, claiming that his father had been an eminent physician in Edinburgh. A man fitting a similar description reportedly approached another witness in the street. The man had asked intuitively whether she was ‘carrying again’, and said that if she were, he knew ‘how to get rid of it’. These accounts indelibly associated the working-class neighbourhoods of Notting Hill in the public imagination as places where illegal abortionists touted for trade, and, relatedly, where sexual deviance and violence thrived.

This evidence, much of it collected but overlooked between 1949 and 1953, assumed a new status in light of growing public and political concern with the nature of the illegal abortion trade. Since the nineteenth century, successive tightening of the law on abortion had delineated the field of competence for health practitioners, and abortion trials became the testing ground for the legal re-enforcement of professional competence in the field of obstetrics and gynaecology. The medical profession was particularly concerned with eradicating competing medical services and ensuring professional autonomy over procedures such as abortion.^40^ By mid-century, safe, therapeutic abortion had in both the medical discourse and the public imagination become firmly associated with the professional, medical practitioner. In 1938, the landmark trial of gynaecological surgeon Aleck Bourne, who successfully defended his decision to perform an abortion on a fourteen-year-old girl who had been raped, established a precedent in case law by which medical practitioners, acting in good faith, could legitimately perform abortions. The legal ruling in this case contributed, as Barbara Brookes and Paul Roth argue, to the medicalization of abortion in that it handed doctors a legal monopoly on abortion decisions, a monopoly that would finally receive statutory sanction in 1967.^41^

Critically, the Bourne trial also perhaps fostered the masculinization of abortion, cementing the image of the safe, professional abortionist as almost exclusively male. While doctors, the majority of whom were men, continued to be implicated in abortion offences following the Bourne ruling, it was ordinary lay women practitioners who were overwhelmingly tried and convicted as abortionists and were the target of the fiercest criticism and public vilification.^42^ In contrast, ‘[th]e physician’, Kate Gleeson argues, ‘was, in fact, often romanticized in the public eye’, even though their services remained legally dubious prior to the passing of the 1967 Abortion Act.^43^ This masculinization of abortion, as Judith Allen notes, may have improved the competitive position of all male abortionists, non-medical included.^44^ Widespread newspaper coverage of the tragic deaths of women at the hands of unskilled abortionists, together with a decline in alternative medical practitioners and a growing public trust in the white-coated physician, would have only increased women's concern with the medical qualifications of their abortionist. By representing himself as medically trained, Christie, so it came to be implicitly feared, had been able to elicit the trust of vulnerable working-class women, for whom a surgical and supposedly safer procedure was beyond their means.

These encounters between Christie, the male imposter-abortionist, and his potential victims exposed other features of the covert world of illegal abortion. The image of Christie touting for business by word of mouth among the cafes and backstreets of Notting Hill revealed an alarming framework within which to evaluate the oral networks and social environments in which abortion transactions were conducted. Yet, in making unsolicited approaches to these women, Christie's behaviour was as much unusual as it was disturbing, in that it did not follow the usual pattern of abortion transactions and it transgressed the public boundaries of intimacy between the sexes. Moreover, the proposition that Christie posed as an abortionist in order to lure his victims spoke to broader concerns about the sexual harassment that thrived in the backstreet abortion trade. It was not unheard of for abortionists to take advantage of the clandestine situation to exploit their vulnerable clients sexually, with some male practitioners offering an abortion in exchange for sexual intercourse.^45^

The Brabin Inquiry also focused on the social networks in and around Ten Rillington Place, in which abortion played a significant role, as contributing to an expanded assessment of the horror of Christie's transgressive behaviour. Historians traditionally viewed abortion as a ‘female subculture’ in which women utilized networks of female relatives, friends and work colleagues to obtain information.^46^ More recently, however, scholars have emphasized heterogeneous networks, male as much as female, within which knowledge about abortion and its methods circulated as an ‘open secret’.^47^ Rillington Place, in several respects, was home to this sort of ‘open secret’; implicitly suggesting that the way abortion was tacitly discussed could leave abortion seekers exposed to unseen dangers. Beryl's pregnancy and her attempts to terminate it were known and discussed by her husband, her immediate family, including her brother and mother-in-law, her friends, and her neighbours, in particular, the Christies. By making an open secret of Beryl's abortion, the Brabin Inquiry gave credence to the idea that abortion had been one of the major determining factors in the circumstances leading to Beryl's death, casting doubt on Timothy Evans's guilt. Furthermore, by showing the way in which Beryl's pregnancy and knowledge about her attempts to ‘get rid of it’ circulated as fact and rumour in and outside of Ten Rillington Place, her murder was transformed into an abortion drama. Beryl's situation highlighted the dangers that pregnant women put themselves in, emphasizing how predatory psychopaths could take advantage of an ‘open secret’ by exploiting informal heterogeneous networks within which the practice of abortion was firmly established.

Brabin's reinvestigation of the forensic evidence, however, continued to rule out abortion as a plausible scenario not only in the circumstances leading to Beryl's death but also in the case of Christie's other victims. Donald Teare, the pathologist in the Timothy Evans trial, defended his original autopsy of Beryl Evans's body. While he reiterated that the bruise on the back of Beryl's vaginal wall was suggestive of an earlier attempt to self-abort, Teare resisted the theory that abortion involving a third party had directly resulted in her death.^48^ Francis Camps, the leading forensic pathologist in the 1953 investigations into the Christie murders, also relegated the abortion angle firmly to the speculative realm of the popular imagination. His continued scepticism which echoed and rearticulated Teare's and his own earlier reluctance to accept the concept of a male backstreet abortionist, led him to entertain the possibility that some of Christie's victims were not only sexually deviant but also willing participants in their own deaths. According to Camps, Christie's victims (with the exception of Beryl) willingly inhaled carbon monoxide as part of a consensual and sexually stimulating encounter with him. Camps interpreted Christie's killing equipment, notably the rubber tubing, not as the surgical kit of an abortionist imposter, but rather something far more sinister relating to the sexual perversion shared by both Christie and his unfortunate female victims.^49^ Thus, socially distanced from both the world of Rillington Place and the fluid and heterogeneous networks of open secrets in which abortion seekers and their families and friends were embedded, neither Camps nor Teare could imagine Beryl as an abortion seeker and Christie as an abortion provider.

## IV

Just as the moral character of Christie's other victims came under critical scrutiny in the Brabin Inquiry, Beryl Evans's character, that of her husband Timothy Evans and of their marriage were negatively evaluated, in a manner that recalled the highly moralized discussions of abortion seekers in trials of illegal abortionists. Thus, despite the greater readiness to contemplate and discuss the abortion issue in the permissive era, portrayals of abortion seekers and the domestic and familial circumstances that shaped their decision to terminate a pregnancy remained profoundly distorted by a reluctance to acknowledge how socio-economic circumstances and poverty shaped working-class decisions about reproduction control.

While the Brabin Inquiry increasingly identified Christie as a potential abortionist and Rillington Place as his place of practice, Beryl was confirmed as typical of the countless women in search of an abortion in post-war Britain. As representatives of the Abortion Law Reform Association (ALRA) regularly pointed out, the large majority of those who asked for abortions were, like Beryl, ‘working-class women who for good reason consider the birth of a child at a given time a threat to the welfare of their home, a burden too heavy for their strengths or their husband's earnings, and a disaster for the children already born’.^50^ Brabin stimulated an interrogation of the material circumstances that precipitated the crime of abortion, asking searching questions about the couple's life together. This retrospective exploration of life in the Evans household, an existence of poverty, hardship and lowering standards in post-war Britain, lent the abortion hypothesis an air of credibility, in part because it exposed the turbulence and insecurity of working-class domesticity, which rendered abortion necessary. However, the Inquiry's lines of investigation mirrored many a typical trial for sexually related crimes, including abortion, in that although it appeared to address the extenuating conditions, it also implicitly focused on the morality of both the defendant and victim.

Returning to the original 1949 statements of John Christie and his wife Ethel, Brabin was reminded that Timothy and Beryl ‘got on very badly together’, with ‘frequent quarrels’ about ‘his lying to her, his associating with other women and financial matters’.^51^ Other witnesses substantiated the Christies' allegation that Timothy had a ‘violent temper’; and that he had, on more than one occasion, assaulted his wife. Lucy Endicott, a friend of Beryl, who it was alleged also had a brief love affair with Timothy, described how she once witnessed Timothy ‘set about her and began hitting her with his hand across the face and body’.^52^ A neighbour, Mrs Hide, gave evidence that she had frequently overheard ‘quarrels and arguments’ between the couple, as did others.^53^

Aside from his violent behaviour, Timothy, it was proposed, had also been a womanizer, accused of having had at least two affairs with other women, which further allowed the Brabin inquiry an interpretative context for understanding why Beryl might have been seeking an abortion. Mrs Probert, Timothy's mother, described the effect of one such attachment on the couple's marriage:

My son and his wife were perfectly happy until August 1949, when a girl, a blonde, who used to work with Beryl … came to live with them. Tim seemed to become very friendly with her and according to Beryl, Tim used to take her to the pictures … In my presence Beryl told (her [the girl]) that she should leave the premises for good as her proper place was with her [Beryl's] husband.^54^

When the woman refused to leave, Beryl reportedly contacted the police, who advised her to go to the West London Magistrates' Court to seek advice and assistance, which she duly did. In the meantime, Tim left the flat with the girl, but returned alone the following night, when he and Beryl ‘made it up’.^55^

Not only was Timothy in possession of a short temper and a wandering eye, but he was also a poor financial provider for his family. Mrs Probert testified that the couple ‘used to have arguments over money’, while Lucy Endicott gave evidence that

Tim used to say that he gave Beryl £5 a week for housekeeping but she used to say she never received £5. He also used to say he was going to work and on some occasions we found out he didn't. He would also make up excuses about not having received his money from Mr. Adler [his employer].^56^

Another witness, a neighbour, described Timothy as an absent husband and father, who was ‘out a good deal’, leaving his wife ‘at home all day with the baby’.^57^ Poorly educated and with learning difficulties, Timothy struggled to hold down well-paid employment; and, it was alleged, often misspent his wages on drink. Timothy was thus cast in the stereotype of those feckless husbands who drive their wives to abortion.

In sum, Timothy's behaviour fell short of the standards of respectable working-class masculinity for this period, a failure that was often used in criminal abortion trials to contextualize why abortion came to be an option for some families but not others. His impaired intelligence and inability to obtain well-paid, stable employment, threatened his home and family with financial insecurity, undermining the ideal of the working-class male breadwinner. Meanwhile, his brutish temper and drunken, adulterous behaviour failed to comply with the emergent post-war ideal of companionable marriage. Violent, drunken, and promiscuous, Timothy was the embodiment of unreformed working-class masculinity, which by the time of the Brabin Inquiry in 1965 would appear even more outmoded.^58^

Despite Timothy's failings as a husband and the violent nature of her own death, Beryl did not receive an altogether sympathetic portrayal in Brabin's reinvestigation of the evidence, which ran parallel to the general denigration of women involved in crimes of a sexual or violent nature, including abortion.^59^ Witness accounts, for instance, suggest that Beryl did not conform to idealized understandings of femininity, particularly in relation to her role as housewife and homemaker. Descriptions of her behaviour and conduct prior to her death could not easily be squared with social expectations of passive femininity and female victimhood.

The seeds of Beryl's supposed feminine failings were sown in Timothy Evans's statements, revisited on day one of the Inquiry. These alleged that Beryl was a poor housekeeper, who ‘was in debt with the rent’ and had fallen behind in the couple's payments for furniture on the hire purchase.^60^ The Inquiry also unearthed statements from family, friends, and neighbours further supporting an image of Beryl as an inattentive, slovenly housewife. When requestioned as to her daughter-in-law's habits, Mrs Probert, disproving of Beryl's wishes to have an abortion, was especially unrelenting in her criticism. According to Mrs Probert, Beryl was ‘dirty in her habits’, ‘did not used to clean the flat’, ‘refused to wash Tim's shirts and socks so that he had to do it himself’, and ‘did not get her husband's meals except on Sundays when they had their only weekly hot meals'.^61^ Previous statements by Mrs Probert and others had cast Beryl as a ‘nagging’ wife, who on occasion deliberately provoked her husband's ire. During one particular altercation between the couple, it was alleged that Beryl had thrown a jar at her husband, injuring his head.^62^ As much as Timothy was a poor provider, in failing to manage the household's meagre finances or to keep a clean and tidy home, Beryl also failed to live up to the expectations of a ‘good’ wife, which again provided further weight to why in this case abortion was a significant dimension. In revisiting these flaws of character, the Brabin Inquiry's lines of investigation played up to a long-standing alarmist assumption held by opponents of abortion that it was dysfunctional, ‘sluttish’ wives, like Beryl, antagonistic to the role of wife and mother, who were the most likely to pursue abortion.

Ultimately, Timothy's reputation was not satisfactorily restored, nor was Beryl's character left untainted, exemplifying both the ambiguity and tensions at the heart of the Brabin Inquiry; and in the legal interrogations of the circumstances in which a working-class woman would seek an abortion. The Inquiry certainly furnished enough circumstantial evidence to establish a link between abortion, Beryl Evans, and John Christie; but in exposing the intolerable state of the Evans's marriage, it inadvertently continued to lend authority to the original judicial decision, that Beryl had died at the hands of her violent husband.

Though the link between abortion and Beryl's death was widely reported in newspaper coverage of the Inquiry and its conclusions, there was nothing by way of editorial commentary on the practice of illegal abortion more generally. Nor does the case appear to have provoked comment from among abortion reformers, despite it having vividly highlighted the potential dangers of deception and sexual exploitation involved in the black market abortion trade. After all, organizations, such as the ALRA, had since the 1930s placed considerable effort into publicizing the tragic loss of life that resulted from backstreet abortion with the hope that the ‘cumulative horror might arouse public conscience to the necessity of clarifying our abortion law’.^63^ From the 1950s, however, the ALRA began to take a more direct approach to law reform through public education and parliamentary lobbying. The focus of the rhetoric of their campaign shifted to include a greater emphasis on highlighting the ‘safety’ of abortion as opposed to its dangers,^64^ and supporting the needs of thousands of ‘desperate women’ unable to secure a therapeutic abortion who deserved a clarification and extension of the law.^65^ Once the Brabin Inquiry was completed, the Evans/Christie case, with its many uncertainties over the nature of Beryl's death, question marks raised over the character of Beryl, and its aura of sexual perversion, was perhaps too unsavoury and sensationalized to be of much use to reformers. Furthermore, as the state-sanctioned Inquiry had failed to exonerate Timothy Evans of his wife's death and in consequence failed to confirm the abortion story, the case was also rendered politically sensitive at a time when the lobby for abortion law reform was reaching its peak. In adding their voice to the chorus of scepticism that greeted the Inquiry's conclusions, abortion reformers would have aligned themselves with popular understandings of the case, which might have risked alienating important conservative political allies.

## V

Brabin judged it more likely than not that Evans did not murder his baby, for which he was hanged, but that he did kill his wife, for which he was not tried.^66^ On 18 October 1966, Timothy Evans was granted a royal pardon, signed by Queen Elizabeth II and Home Secretary Roy Jenkins, and was reburied away from Pentonville Prison, in a Roman Catholic churchyard. However, Rillington Place continued to be the focus of public attention, despite its being renamed Ruston Place in 1954. The 1971 film *10 Rillington Place*, based on Kennedy's book, was shot in a house just two doors down from number 10, as the house itself was not practical for filming. This film helped to secure in the popular imagination that it was not Timothy Evans, but rather John Christie who was responsible for the death of Beryl Evans. By way of a conclusion, we now want to return to how the film, in many respects, serves as yet another site in which Ten Rillington Place and the world of backstreet abortions were rewoven to form a new narrative following the liberalization of abortion law.

Richard Fleischer's biopic confidently focused on the days leading up to Beryl Evans's death, providing a cinematic lens through which to view the daily dysfunction and impoverished life of post-war Britain, constituting an attempt to readdress events surrounding her end. Divested of the potent legal and political concerns which formed the backdrop to the case in the 1950s and 1960s, the film provided a sympathetic portrayal of Beryl and Timothy. The Brabin Inquiry had figuratively put the couple on trial for their role in bringing about the horrors of Ten Rillington Place, and had ultimately not revised Timothy Evans's connection to the death of his wife. The film, by contrast, enabled a complete, meticulous retelling of events at Rillington Place. It restores in many ways the moral character of Timothy and Beryl, presenting them both (along with their daughter Geraldine) as the unfortunate victims of a darker, more twisted figure – John Christie. Portrayed as a ‘decent’ yet struggling young working-class couple, Timothy and Beryl are culturally reimagined, rendered as effective articulations of how imagined Victorian forces and values had imprisoned Britons, especially the younger generation. Christie, gaining coherence in the new era, is firmly recast as a powerful monstrosity for a shifting zeitgeist without the fierce legal and political contestations that had surrounded the case in the previous decades.

In the wake of both Brabin and the 1967 Abortion Act, the film retrospectively inspects John Christie's relationship with the Evanses, which meant exploring the abortion encounter between Christie and Beryl in meticulous and vivid detail. The ‘operation scene’, described at the outset of this article, invokes a whole series of social, material, and cultural associations entwined with the experience of, and worries over, illicit abortion practices: images imbued with meanings that would have been immediately recognizable to an audience still familiar with the backstreet abortionist. Consider, for example, the scene leading up to Beryl's death, when Christie prepares for the ‘operation’. He makes Beryl a cup of tea to settle her nerves, calling into play awareness of the close intimacy and domesticity involved in an illegal abortion.

Critically, for our purposes, even before this scene takes place, the film ensures a frank, candid discussion of reasons for an abortion, positioning the modern viewer in sympathy with why a woman might terminate a pregnancy, and the injustice of repressive legislation. For example, Beryl even explains to a female friend that an abortion is her ‘right’, thereby asserting ownership of her body. With abortion now legal, and given the subsequent shift in the language of abortion in terms of rights and freedom in the context of the then burgeoning women's liberation movement, this cinematic retelling of events served to condemn those socially conservative attitudes that had held back abortion law reform. The cocktail of conservative morality and repressive legislation created opportunities for illegal abortionists, placing married and unmarried women in situations that carried countless risks to their lives, as well as being sexually exploitable scenarios, as powerfully demonstrated by events inside Rillington Place. In this way, the 1971 film recast Christie and his killing of Beryl Evans as an emblematic, cautionary tale of what could become the fate of pregnant women who, out of desperation, are driven into the hands of an unorthodox practitioner who may have no intention of performing an abortion.

More broadly, as we have argued throughout, engaging with the changing profile of backstreet abortion practices and their historically specific inscriptions, meanings, and appropriations, enables us to develop a historical understanding of the case of Ten Rillington Place. It opens up a fresh direction for pursuing integration of the broader historical pattern of gender relations, socio-cultural, medico-legal, and political dynamics of modern Britain. Exploring both the presence and absence of abortion discourse in this specific location and murder case provides us with a historical framework within which to reassess abortion in terms of the practice and the (often fragile and ambiguous) meanings it held, not only for the narrower concerns of reproductive control, but also in its association with the broader issues of family life, sexuality, and gender relations, and with the wider political climate of post-war Britain.

The plethora of readings of Ten Rillington Place, with their diverse and shifting attitudes towards abortion and the rich potential of the male abortionist for signification, provide insights into how there had existed a powerful and persistent societal blind spot on abortion at the time of both the Evans and Christie trials. This blind spot was crucially so strong as to have effectively distorted the criminal investigations and the trials themselves, reflecting a general reluctance to come to terms with the concept of the male backstreet abortionist. Only when public acceptance of the case for legalizing abortion – and entrusting it to the monopoly expertise of a largely male medical profession – grew in the more liberal sexual climate of the 1960s and beyond did the revisionist argument of Ludovic Kennedy, highlighting the significance of abortion in the episode, gain a sustained hearing. The greater readiness to contemplate and discuss the abortion issue in the permissive era made it possible posthumously to pardon Evans, despite the ambiguous outcome of the Brabin inquiry. Yet even in the permissive era, portrayals of abortion seekers remained deeply ambiguous, revealing an unwillingness to acknowledge how socio-economic circumstances and poverty shaped working-class decisions about reproduction control. Our modern-day understanding of the innocence of Timothy Evans in the murder of his wife, then, became entrenched in the 1970s, as one that recognized a significant shift in public and political sensibilities towards abortion following its legalization.

## Footnotes

1 *10 Rillington Place* (Dir. Richard Fleischer, 1971).

2 The John Christie murders have a very threadbare historiography which is beset by elaboration and sensationalism. Much of the secondary literature is biographical and focuses on establishing the degree of guilt or innocence of John Christie for the murder of Beryl Evans. See J. Eddowes, The two killers of Rillington Place (London, 1994); E. F. Gammon, A place to remember: 10 Rillington Place (London, 2012); E. Marsten, John Christie (London, 2007); J. Oates, John Christie of Rillington Place: biography of a serial killer (Barnsley, 2012); N. Root, Frenzy! How the tabloid press turned three evil serial killers into celebrities (London, 2012). Until recently, there has been a notable lack of critical analysis. The most credible work on the Rillington Place murders of recent years has been produced by Frank Mort. Mort uses the Christie case to illuminate the ways in which homicide cases can be a discursive expression of cultural, moral, and social geography. His approach counters prevailing assumptions of violent crime as ahistorical, and stimulates new ways of thinking about the murderers, crime scenes, and victimhood. F. Mort, Capital affairs: London and the making of the permissive society (New Haven, CT, and London, 2010). For a historical exploration of the forensic aspects of the case, see I. Burney and N. Pemberton, ‘The house of murder: the Christie investigation and the making of the modern crime scene’, Manchester Memoirs, 150 (2013), pp. 108–23.

3 G. Davis, ‘The medical community and abortion law reform: Scotland in national context, c. 1960–80’, in I. Goold and C. Kelly, eds., Lawyers' medicine: the legislature, the courts and medical practice, 1760–2000 (Oxford, 2009), pp. 143–65; G. Davis and R. Davidson, ‘“The fifth freedom” or “hideous atheistic expediency”: the medical community and abortion law reform in Scotland, c. 1960–75’, Medical History, 50 (2006), pp. 29–481650287010.1017/s0025727300000120PMC1369012; E. L. Jones, ‘Representations of illegal abortionists in England, 1900–1967’, in A. Mangham and G. Depledge, eds., The female body in medicine and literature (Liverpool, 2011), pp 196–200; see also, M. Arnot, ‘Infant death, child care and the state: the baby–farming scandal and the first infant life protection legislation of 1872’, Continuity and Change, 9 (1994), pp. 271–311.

4 *Vera Drake* (Dir. Mike Leigh, 2004).

5 J. Allen, ‘The trials of abortion in late nineteenth- and early twentieth-century Australia’, Australian Cultural History, 12 (1993), pp. 87–99, at p. 89.

6 See F. Tennyson Jesse's A pin to see the peepshow (London, 1934), in which the character Julia, based on Edith Thompson, undergoes an illegal backstreet abortion.

7 A. Ballinger, Dead woman walking: executed women in England and Wales, 1900–1955 (Aldershot, 2000), pp. 5–6. For historical analysis of the Thompson-Bywaters trial and the role of abortion, see L. Bland, ‘The trials and tribulations of Edith Thompson: the capital crime of sexual incitement in 1920s England’, Journal of British Studies, 47 (2008), pp. 624–48, at p. 641.

8 Jones, ‘Representations of illegal abortionists’, p. 200; Allen, ‘The trials of abortion’, pp. 89–90.

9 Our narrative description of the police investigation of the murder of Beryl Evans and later the Christie murders is drawn from The National Archives (TNA), CAB 143, Inquiry into case of Timothy John Evans (Brabin Inquiry): transcripts and papers.

10 R. v. John Timothy Evans, trial transcripts of shorthand, TNA, CAB 143/11.

11 Jones, ‘Representations of illegal abortionists’, pp. 207–8.

12 R. v. John Timothy Evans, trial transcripts of shorthand, TNA, CAB 143/11.

13 Ibid.

14 ‘Reports of societies: criminal abortion’, *British Medical Journal*, 2, 3698 (1931), pp. 946–7.

15 D. Teare, ‘Air embolism in criminal abortion’, Lancet, 244 (1944), p. 242; D. Teare, ‘Medico-legal aspects of criminal abortion’, British Medical Journal, 1, 4764 (1952), pp. 915–171491619810.1136/bmj.1.4764.915PMC2023361; D. Teare, ‘The post-mortem appearance in fatal procured abortion’, Medicine, Science and the Law, 4 (1964), pp. 177–910.1177/00258024640040030614184181.

16 Police statement of Ethel Christie to the police officers investigating Beryl and Geraldine Evans's deaths in December 1949, TNA, CAB143/44.

17 R. v. John Timothy Evans, trial transcripts of shorthand, TNA, CAB 143/11.

18 For a comprehensive review of the scientific and medical aspects of the case, see F. Camps, Medical and scientific investigations in the Christie case (London, 1953).

19 Police statement of John Christie in F. Tennyson Jesse, ed., Trials of Timothy John Evans and John Reginald Halliday Christie (London, 1957), p. 161.

20 Camps, *Medical and scientific investigations in the Christie case*, pp. 135–55.

21 Mort, *Capital affairs*, p. 129.

22 Report by chief inspector (CID) on the Christie case, TNA, CAB 143/24.

23 This emerged at the Brabin Inquiry in 1965; see transcripts of witness testimony, TNA, CAB 143/31.

24 J. Walkowitz, Prostitution and Victorian society: women, class and the state (Cambridge, 1980), p. 80.

25 Margaret Wilson was approached by Christie who reportedly asked her whether she was pregnant; see report by chief inspector (CID) on the Christie Case, TNA, CAB 143/24.

26 R. v. John Reginald Halliday Christie, transcript notes of shorthand, TNA, CAB 143/13.

27 Ibid.

28 Report of an inquiry into certain matters out of the deaths of Mrs Beryl Evans of Geraldine Evans and out of the conviction of Timothy Evans of the murder of Geraldine Evans (London: Home Office, July 1953), p. 15.

29 K. Hindell and M. Simms, Abortion law reformed (London, 1971); B. Brookes, Abortion in England (London, 1988); E. L. Jones, ‘Attitudes to abortion in the era of reform: evidence from the Abortion Law Reform Association correspondence’, Women's History Review, 20 (2011), pp. 283–9810.1080/09612025.2011.55632321751480.

30 ‘Up the junction’ for *The Wednesday Play*, broadcast on the BBC on 3 Nov. 1965; Nell Dunn, *Up the junction* (London, 1963).

31 S. Brooke, Sexual politics: sexuality, family planning, and the British left from the 1880s to the present day (Oxford, 2011), p. 158.

32 M. D. Kandiah and G. Staerck, eds., The Abortion Act, 1967 (ICBH Witness Seminar Programme, 2002). In her evidence to the witness seminar, ALRA activist Diane Munday commented: ‘that issue of *Dr Finlay's Casebook* moved the campaign on three years for us without us doing anything. It raised the issue in a way we were not able to’, p. 56.

33 Kennedy alleges that Rita's employer at the Lyons tea shop made introductions to the Samaritan Hospital for Women upon learning of Rita's condition, but that Rita never turned up to discuss her confinement. L. Kennedy, 10 Rillington Place (London, 1961), pp. 216–19.

34 Ibid., p. 217.

35 Ibid., p. 218.

36 S. Brooke, ‘“A new world for women”? Abortion law reform in Britain during the 1930s’, American Historical Review, 106 (2001), pp. 431–5918186164.

37 A. Jenkins, Law for the rich (London, 1964), p. 36.

38 Kennedy, *10 Rillington Place*, pp. 64–5.

39 Ibid., p. 50.

40 J. Keown, Abortion, doctors and the law: some aspects of the legal regulation of abortion in England from 1803 to 1982 (Cambridge, 1988), ch. 3.

41 B. Brookes and P. Roth, ‘R. v. Bourne and the medicalisation of abortion’, in M. Clark and C. Crawford, eds., Legal medicine in history (Cambridge, 1993), pp. 314–43.

42 On the typical accusations made against lay women abortionists, see M. Woodside, ‘The woman abortionist’, J. G. Weir, ‘Lay abortionists’, and J. Peel, ‘Attitudes in Britain’, in Family Planning Association, *Abortion in Britain: proceedings of a conference held by the Family Planning Association at the University of London Union* (London, 1966), pp. 35–8, 39–42, and 67–9.

43 K. Gleeson, ‘Persuading parliament: abortion law reform in the UK, Australiasian Parliamentary Review, 22 (2007), pp. 23–42, at p. 27.

44 J. Allen, Sex and secrets: crimes involving Australian women since 1880 (Melbourne, 1990), pp. 166–7.

45 Weir, ‘Lay abortionists’, p. 40; L. J. Reagan, When abortion was a crime: women, medicine, and the law in the United States, 1867–1973 (Berkeley, CA, 1997), p. 199; Allen, ‘Trials of abortion’, p. 89; C. Usborne, Cultures of abortion in Weimar Germany (Oxford, 2007), p. 89.

46 See P. Knight, ‘Women and abortion in Victorian and Edwardian England', History Workshop Journal, 4 (1977), p. 57–6910.1093/hwj/4.1.5711610301.

47 Reagan, *When abortion was a crime*, p. 45; see also K. Fisher, Birth control, sex and marriage in Britain, 1918–1960 (Oxford, 2006); Jones, ‘Attitudes to abortion’, p. 295.

48 Evidence of Donald Teare, TNA, CAB 143/32.

49 Evidence of Francis Camps, TNA, CAB 143/37.

50 Janet Chance, chairman of ALRA, cited in Brooke, ‘“A new world for women”’, p. 448.

51 Statements of John and Ethel Christie, TNA, CAB143/31.

52 Evidence of Lucy Endicott, TNA, CAB 143/32.

53 Evidence of Mrs Hide, TNA, CAB/143/33.

54 Evidence of Mrs Probert, TNA, CAB143/38.

55 Evidence of Mrs Probert, TNA, CAB143/38.

56 Evidence of Mrs Probert and Lucy Endicott, TNA, CAB143/32.

57 Evidence of K. B. Alexander, TNA, CAB143/43.

58 S. Brooke, ‘Gender and working-class identity in Britain during the 1950s’, Journal of Social History, 34 (2001), pp. 773–951806477910.1353/jsh.2001.0043.

59 On the public condemnation of the private lives of women involved in crime see, Ballinger, *Dead woman* *walking*, and S. D'Cruze and L. Jackson, eds., Women, crime and justice in England since 1660 (Basingstoke, 2009).

60 Statement of Timothy Evans, TNA, CAB143/31.

61 Evidence of Mrs Probert, TNA, CAB143/32.

62 Evidence of Mrs Probert, TNA, CAB143/32.

63 A. Jenkins, ‘Back Street Surgery’, Lancet, 251 (1948), p. 789.

64 Gleeson, ‘Persuading parliament', pp. 31–2.

65 See Abortion Law Reform Association, *In des**peration: letters sent to the Abortion Law Reform Association* (Burlington Press, Foxton, England, no date), pamphlet available in Wellcome Library, Archives and Manuscripts, SA/ALR/D2; for further discussion of ALRA campaign literature in this period see Jones, ‘Attitudes to abortion’, pp. 289–90.

66 *Report of an inquiry by the Hon. Mr. Justice Brabin into the case of Timothy John Evans* (London, 1966).

